# Hearing loss and vestibular schwannoma: new insights into Schwann cells implication

**DOI:** 10.1038/s41419-023-06141-z

**Published:** 2023-09-23

**Authors:** Tasnim Mohamed, Valentina Melfi, Alessandra Colciago, Valerio Magnaghi

**Affiliations:** 1 Department of Pharmacological and Biomolecular Sciences “Rodolfo Paoletti”, Università degli Studi di Milano, Via G. Balzaretti 9, 20133 Milan, Italy; 2 Fondazione IRCCS Istituto Nazionale dei Tumori, Via G. Venezian 1, 20133 Milan, Italy

**Keywords:** Mechanisms of disease, Oncogenesis

## Abstract

Hearing loss (HL) is the most common and heterogeneous disorder of the sensory system, with a large morbidity in the worldwide population. Among cells of the acoustic nerve (VIII cranial nerve), in the cochlea are present the hair cells, the spiral ganglion neurons, the glia-like supporting cells, and the Schwann cells (SCs), which alterations have been considered cause of HL. Notably, a benign SC-derived tumor of the acoustic nerve, named vestibular schwannoma (VS), has been indicated as cause of HL. Importantly, SCs are the main glial cells ensheathing axons and forming myelin in the peripheral nerves. Following an injury, the SCs reprogram, expressing some stemness features. Despite the mechanisms and factors controlling their biological processes (i.e., proliferation, migration, differentiation, and myelination) have been largely unveiled, their role in VS and HL was poorly investigated. In this review, we enlighten some of the mechanisms at the base of SCs transformation, VS development, and progression, likely leading to HL, and we pose great attention on the environmental factors that, in principle, could contribute to HL onset or progression. Combining the biomolecular bench-side approach to the clinical bedside practice may be helpful for the diagnosis, prediction, and therapeutic approach in otology.

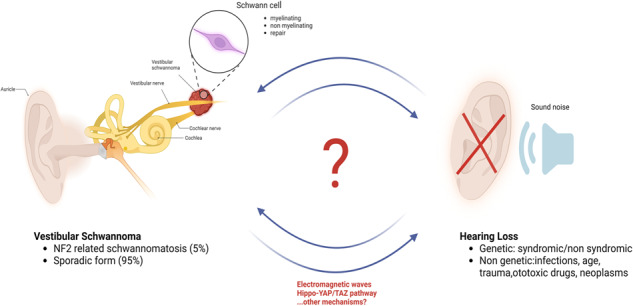

## Introduction

Hearing loss (HL) is one of the most common sensorineural impairments in humans and a heterogeneous disorder with a complex etiology (deeply reviewed in refs. [1, 2]). Many factors have been indicated to cause HL, among which can be considered the genetic modifications (syndromic and non-syndromic), the degeneration of sensory neurons (i.e., the spiral ganglion neurons, SGNs) or hair cells (HCs) in the auditory pathway and the demyelination along the cochlear nerve [3]. Overall, the causative genes are classified as presynaptic, synaptic, and postsynaptic [4]. Congenital cytomegalovirus infection, instead, is the most common non-genetic cause of sensorineural HL (SNHL) among children [5]. Similarly, age-related HL (i.e., presbycusis) is the most frequent type of SNHL affecting 40–50% of the population by age 75 [6], although also traumatic injuries, for instance, following a fierce noise exposure, or the exposure to ototoxic drugs (e.g., gentamicin, streptomycin or kanamycin) can lead to HL. In addition, a variety of nervous neoplasms can cause HL, including the vestibular schwannoma (VS), described below.

Normal hearing requires anatomical integrity and functional compliance of all parts of the auditory system: the outer, middle, and inner ear, the cochlear nerve as well as the central auditory pathways [6]. Therefore, based on the anatomical morphology and according to the affected site, HL can be conductive, sensorineural, central, or psychogenic [6, 7].

**Fig. 1:**
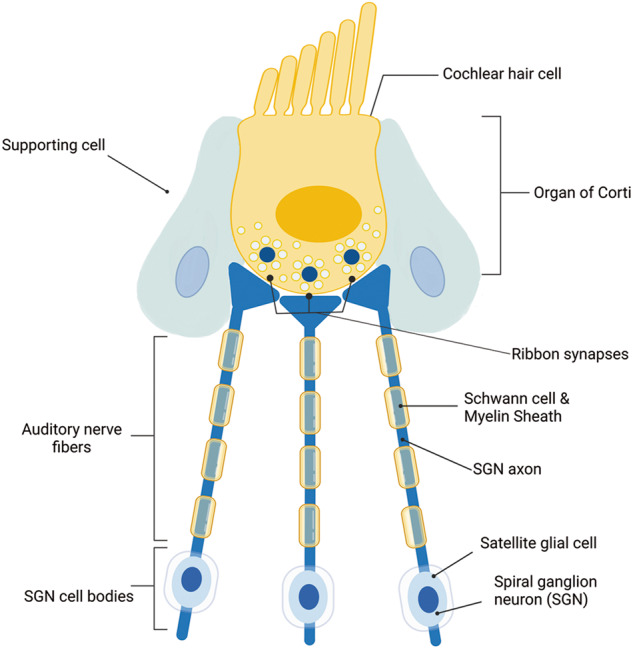
Schematic representation of main cells forming the auditory structure in the organ of Corti, including the afferent fibers and the glial cells.

In the cochlea, acoustic stimuli are detected by the HCs in the organ of Corti, which transduce the acoustic signals to the primary auditory SGNs through the ribbon synapses (see Fig. 1). In particular, HCs can be divided in inner HCs (IHCs), bearing the K+ mechanosensitive channels which opening initiates the auditory impulse, and the outer HCs (OHCs), in contact with the tectorial membrane and deputed to modulate and amplify the auditory signals. Primarily, IHCs transmit the stimulus to the SGNs, which can be classified into two types [8]. Type I SGNs, which comprise 90–95% of the SGN population, are covered by satellite cells, and 10–20 of these neurons extend single, unbranched, myelinated neurites [i.e., ensheathed by Schwann cells (SCs)] to the organ of Corti, where they contact with IHC. Type I SGNs can also be divided into three different populations named type Ia, type Ib, and type Ic on the base of the transcriptional profile [9, 10]. Similarly, type II SGNs (accounting for 5–10% of total SGNs) project thin and unmyelinated axons to multiple OHCs [8]. Central processes of the SGNs form the auditory nerve, are ensheathed by oligodendrocytes and project to specific areas in the brain. In addition, HCs are surrounded by glial-like supporting cells, which share many molecular and physiological features with the glial cells of the central nervous system (CNS). These cells, which were thought to be silent and only mechanically supporting HCs, were recently reconsidered as actively playing important roles in auditory transduction [11]. Glial-like supporting cells also participate in the maturation, proliferation, protection, and regeneration of cochlear cells [12, 13].

Basically, the correct function of HCs and SGNs is fundamental for normal hearing and communication, so much that an alternation or degeneration of either components, due to the causes previously described (i.e., genetic disorders, viral infections, loud noise, ototoxic drugs, and aging) give SNHL [14]. In particular, HC loss or synaptic degeneration are both signs of HL, representing the preponderant cause of SNHL. Damage or loss of IHCs can produce auditory deficits even when thresholds remain unchanged. Migration, differentiation, and survival of SGNs, instead, is largely dependent on surrounding SCs [15]. Therefore, myelin is essential for the rapid and saltatory conduction of auditory signals, on which acoustic coding is depending [16]. In particular, the myelin sheath morphology appears highly tuned to mediate the auditory action potential timing [17], to the point that demyelination of the cochlear nerve causes HL.

**Fig. 2:**
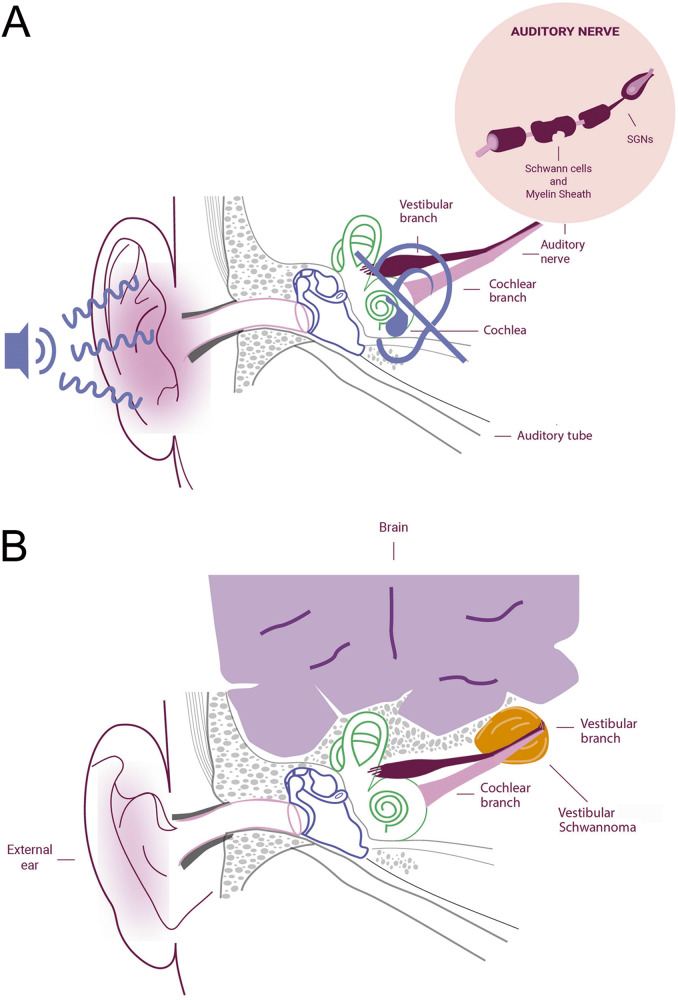
Causes of hearing loss (HL) and vestibular schwannoma (VS). **A** Illustration of HL and demyelination of Schwann cells (SCs). Among many different etiologies of sensorineural HL (SNHL), the extensive loss of the myelinated axonal processes in the auditory nerve may be followed by alteration or degeneration of hair cells (HCs) and spiral ganglion neurons (SGNs). The resulting degenerative process reduces the neuronal capability in initiating and propagating action potentials in response to electrical stimuli, leading to nerve conduction vulnerability. **B** Anatomical illustration of the internal ear structures affected by VS (also called acoustic neurinoma).

The first step of the pathologic process leading to SNHL, with SGN degeneration, entails a rapid and extensive loss of the unmyelinated axonal processes within the organ of Corti [18], followed by a gradual degeneration of the myelinated portion of the peripheral axons (within the osseous spiral lamina), and of the SGN soma inside the Rosenthal’s canal [8, 19] (see Fig. 2A). Therefore, the degenerative process determines a reduction of the number of myelin lamellae surrounding the SGNs axons [18]. The resulting SCs demyelination determines an increase of the neural membrane capacitance [20], reducing the capability of a neuron in initiating and propagating action potentials in response to electrical stimuli, and leading to a delayed nerve excitation. The rate of action potential propagation is reduced, concomitantly with an increase in the nerve conduction vulnerability [21]. Moreover, the demyelination rises the refractory properties of the nerve [20], determining a conduction block in SGNs [22].

Mice exposed to an injury, such as loud or chronic noise, showed electrophysiological signs of nerve damage with striking demyelination of the axons, thinner myelin sheath and larger internodal distance [23]. Following a damage, the HCs of the vestibular/cochlear epithelium go towards an adaptive cellular reprogramming, triggering the direct conversion of neighboring glial-like supporting cells into new HCs [24–26]. As reported above, this process shows similarities with the injury-induced SCs reprogramming. Interestingly, the generation of cochlear HCs is dependent by Wnt signaling and is strongly enhanced when Notch signaling is inhibited [27]; these two important factors are also involved in the SCs radial sorting and development (see below), respectively.

Differently from the classic forms of HL, hidden hearing loss (HHL) is an auditory neuropathy characterized by reduced suprathreshold amplitude of the sound-evoked auditory nerve action potential [28–30]. HHL is primarily a synaptopathy of the IHCs, leading to a defective neurotransmission and presenting alterations in the ratio of the waveform peaks generated by HCs [31]. The ribbon synapses are very sensitive to noise, which induces cochlear damage likely beginning at the IHCs synapse, then progressing to loss of HCs. However, participation of SCs in the pathogenesis of HHL has been recently suggested [32].

### Schwann cells

SCs are the main glial cells of the peripheral nervous system (PNS). Their function is principally the formation of the myelin sheath, which isolates axons allowing the saltatory conduction of the action potential [33]. However, SCs role is multifaceted and complex, given their participation in different processes spanning from the PNS development [34] to the nerve myelination and repair [35]. SCs are derived from neural crest cells, through different developmental stages, including SC precursors, immature SCs, pro-myelinating SCs, until the formation of mature myelinating and/or nonmyelinating SCs [33, 36]. The mechanisms governing the development of SCs precursors entail the activation of some transcription factors, like Sox10, Krox20 and Pax3 [37, 38]. In general, these steps arise from a strict balance between cell proliferation, migration, and differentiation, under the control of adhesion proteins, growth factors, hormones and neurotransmitters [33]. Therefore, SCs have a certain grade of plasticity, assuming different cell stages depending by the physio-pathologic *mileau* that regulates the SCs-neuron crosstalk. In the developing PNS, SCs precursors comigrate with axon elongation, depending by axonal signals [39]. In this context, the NRG-1/ErbB2/3 signaling system [40] and the Notch signaling [41] are determinants of SC precursor development into immature SCs, which are then dependent on autocrine signaling for survival [42]. In addition, immature SCs enter in contact with large axons proceeding towards the pro-myelinating and myelinating phenotype, while the SCs facing the small caliber axons differentiate to the nonmyelinating phenotype, leading to the formation of Remak bundles [34]. This process, called “radial sorting”, contributes to build the morphological aspect of the mature nerve, in which the SCs myelinate only one axon segment in 1:1 relationship, while the nonmyelinating SCs ensheath some small axons simultaneously, without forming the myelin [34]. From a neurophysiological point of view, the myelinated axons constitute the motor fibers, whereas the lightly myelinated or the unmyelinated axons comprise the sensory, mechanosensitive or nociceptive fibers. Radial sorting is mostly controlled by SCs-derived signals, such as laminins, dystroglycan and integrins, which regulate the extracellular matrix and establish the SCs polarization [34]. Even though few axonal molecules seem to be implicated in radial sorting, for instance, Wnt is expressed on axons and may signal to SCs to regulate their sorting [43]. Among the signaling pathways activated for the SCs myelination, the essential role of cAMP was first hypothesized early in the ’80–’90, when it was demonstrated that cAMP levels regulate the expression of myelin transcription factors and proteins (e.g., the myelin protein zero (P0) or the myelin basic protein (MBP)), controlling the progression of SCs toward the myelinating phenotype [44, 45].

SCs are involved in the pathogenesis of several dysmetabolic, traumatic and hereditary diseases affecting the PNS, such as different forms of the most common Charcot–Marie–Tooth (CMT) disease [46]. In general, SCs are plastic and remodel the nerve during life, with a certain grade of periodicity even slow [47]. After a nerve damage, however, the mature SCs reprogram and dedifferentiate from the myelinating or nonmyelinating state into the so-called “repair SCs”, then supporting the nerve regeneration. Despite c-Jun has been indicated as the main transcription factor regulating the repair SCs [48], other factors are involved; the SCs characteristics as well as the changes occurring during the transition from mature to repair SCs are described in detail elsewhere [48]. Assuming this phenotype, SCs recover a proliferating and migrating condition and express some stemness features, undergoing a process of partial epithelial-to-mesenchymal transition (EMT) [49]. EMT is a biological phenomenon by which cells lose their differentiated characteristic and acquire mesenchymal features [50], although the EMT program frequently run through hybrid cell stages, which on balance constitute the so-called partial EMT process [51]. Notably, the EMT is frequently associated with activation of genes typical of stem cell states and increased stemness, like Snail, Slug or TGFβ [52, 53], and it seems to contribute to the physio-pathologic process of tumorigenesis [54]. In accordance, the SCs have been implicated also in the origin of some kinds of tumors [55], likely through a partial EMT process. Recent evidence confirmed the crosstalk between PNS, nerves and tumor microenvironment, pointing out the importance of precursors SCs in supporting tumor onset and progression [56–58]. In pancreatic and colon cancer, the SCs dedifferentiate from the nerves surrounding the tumor and are chemoattracted toward the cancer cells, in turn stimulating the tumor progression and invasion [59].

In SCs, the adaptive cellular reprogramming ordinarily occurring following a PNS damage, but also in tumorigenesis and cancer progression, shows similarities to the injury-induced changes which happing in cells of other tissues, such as pancreas, liver, skin, but also in the vestibulocochlear epithelium of the ear. In this light, in some instances, it was assumed that SCs in the injured acoustic nerve can re-differentiate in regenerative cells, although their clear involvement in the pathogenesis of HL has not been so far investigated and is matter of discussion in this review.

### Role of Schwann cells in hearing loss

As reported above, SCs alterations in the auditory system have been hypothesized as one of the possible causes of HL or HHL. In this light, demyelination of SCs represents the main morphologic damage of the auditory nerve, bearing to a conduction block [22]. The SCs surrounding the SGNs, indeed, mostly myelinate these neurons and are a source for the neurotrophic support (i.e., producing growth factors like brain derived neurotrophic factor, BDNF, or neurotrophin-3, NT-3) of SGNs [15]. Thereby, a functional auditory nerve relies on a healthy population of SCs producing neurotrophic factors, so much that a SCs detriment leads to a lack of neurotrophic support for the SGNs

The neurotrophins, nerve growth factor (NGF), BDNF and NT-3, exert their activity through the interaction with a group of receptors including the p75 low-affinity neurotrophin receptor (p75NTR) and the tyrosine kinase receptors TrkA, TrkB and TrkC; while NGF predominantly binds to TrkA, BDNF binds to TrkB. In the postnatal SGNs the receptor p75NTR is usually undetectable, albeit some studies showed that, post-deafening, it become expressed in the SCs surrounding the SGNs [60, 61]. During deafening and loss of cochlear HC, indeed, the SCs enter a tightly regulated system of cell proliferation and apoptosis, so that p75NTR participates in either these cellular responses. It was hypothesized that p75NTR might have a role in maintaining a population of SCs, necessary for the functioning of the auditory nerves after deafening [61].

On the whole, SCs modify their physiologic state responding to other growth factors, including the fibroblast growth factor (FGF). To ascertain that FGF plays a role also in the embryonic development of the cochlea and in the maintenance of normal auditory function, targeting SCs, a study was performed in 2’,3’-cyclic nucleotide 3’-phosphodiesterase (CNP)-driven conditional FGF receptor 1/receptor 2 double knockout mice [62]; given that CNP is primarily expressed in SCs and myelin, this method allowed the specific repression of FGF-mediated actions in SCs. Mutant mice exhibited a significant loss of myelinated SGNs in adults, accompanied by age-dependent hearing impairment [3]. This effect was determined by in an attenuation of the trophic support to neurons by glial cells, indicating that “FGF signaling” exerts important effects in the SCs surrounding the SGNs.

Interestingly, the involvement of SCs, even indirectly, in the pathogenesis of HL is suggested also by the observation that some classic peripheral myelin disorders lead to auditory neuropathy. More than 36 genes [e.g., coding for the specific SCs myelin proteins as P0, peripheral myelin protein of 22 kDa (PMP22), connexin of 32 kDa (Cx32), or the transcription factor Sox10], are involved both in the inherited forms of peripheral neuropathy and in HL [63]. For instance, a number of patients with the Guillan-Barre syndrome (a group of immunomediated peripheral neuropathies) showed abnormal auditory responses, consistent with a permanent auditory neuropathy [64, 65]. Similarly, signs of classical auditory deficits have been reported in patients with the common spectra of CMT neuropathies, although the auditory phenotype was quite variable [66, 67]. CMT is a group of clinically and genetically heterogeneous neuropathies [68]. The most common type 1 CMT (CMT1) is a demyelinating form affecting primarily the SCs, presenting morphological signs of myelin degeneration and slowing of motor conduction velocity. Among CMT1, the CMT1A is due to a duplication of PMP22 gene, bearing to toxic protein overexpression and SCs impairment [69]; CMT1A is responsible for more than 60% of all CMT neuropathies. The type 2 CMT (CMT2), instead, is the axonal form affecting the motor, the sensory or both fibers, but presenting less neurophysiological signs of conduction velocity impairment (conduction velocity more than 38 m/s) [68]. More than 60% of CMT1 and more than 85% of CMT2 subjects suffer of impaired processing of auditory temporal cues and/or abnormal speech understanding [67]. A case of sudden, profound and bilateral SNHL has been described for a patient with CMT2 disease [70], further raising the attention on HL as a potential complication of CMT diseases. Other cases of subjects with CMT that presented absent brainstem auditory evoked potential (BAEP), likely for demyelination of auditory pathways, were reported [71]. Likewise, the so-called “X-linked form” of the demyelinating CMT disease (CMT1X), that is due to a mutation in the connexin Cx32, leads to HL [72].

Overall, the variability in peripheral neuropathy phenotypes highlights the critical role of SCs in myelination of the auditory system as well as in long-term survival and function of the auditory nerve. In this context, the transient SCs demyelination of the auditory nerve leads to a permanent disruption of terminal nerve heminodes and auditory deficits, which are characteristic signs of the HHL [32]; this mechanism has been proposed as a new pathologic cause for HHL [32]. In particular, transient loss of cochlear SCs results in permanent auditory deficit because of rapid auditory nerve demyelination, without alterations in synaptic density, but rather correlating with a specific and permanent degeneration of the first heminodes at the auditory nerve axon, close to the IHCs. Transient SCs ablation significantly reduces the suprathreshold amplitude of the auditory response; at the same time, even the suprathreshold latencies and the conduction velocity of the auditory nerve are affected [32]. Interestingly, a paper by Choi et al. [73] reported that CMT1A patients showed normal hearing threshold levels and speech perception score, in a quiet background. Conversely, CMT1A subjects had significant decrease of speech perception in noisy background, correlating with alteration in the temporal auditory processing. Therefore, it was argued that in CMT1A patients the demyelination of the auditory nerve causes defective cochlear neurotransmission, which can be considered a type of HHL. Remarkably, the reduction of the temporal resolution test can be used as an additional marker of CMT1A disease [73]. However, almost all the studies on the role of SCs in HL were addressed to evaluate the myelinating SCs of the cochlear/auditory system, whereas the nonmyelinating SCs (covering unmyelinated axons of type II SGNs, directed to OHCs) seems poorly considered and would deserve further investigation.

### Therapies for hearing loss

Current therapeutic approaches for HL (see Table 1) varies according to individual cases and generally include hearing aids or cochlear implants. The outcomes of cochlear implantation are normally satisfactory, whereas this approach results dependent by the site of lesion and is largely ineffective in subjects with auditory nerve alterations [2]. However, these approaches target, as a rule, patients with profound hearing loss and are not appropriate for subjects with mild HL or HHL, as the substantial amount of people with CMT diseases.

**Table 1:** Current and proposed therapeutic approaches for HL.

Hearing loss		
Therapy	Target	Possible mechanism
Cochlear implant	Inner ear/cochlea	
Hearing aids - Removable hearing aid - Analogue hearing aid - Digital hearing aid	Inner ear	
Stem cell-based therapy	Inner ear cells	Cellular differentiation of stem cells into HCs and SGNs

Presently, the stem cell-based therapy is an option even more realistic, with increased interest (reviewed in ref. [74]). Significant progress was achieved from the cellular differentiation of stem cells, likely pluripotent stem cells (PSCs), into HCs and SGNs [75–77]. In some cases, this approach may rely on the supportive integration with drugs and/or growth factors, improving neuronal elongation and synaptic connections [78]. However, the use of growth factors might be generally controversial and should be finely tuned because some might promote tumoral growth [79–81]. Moreover, stem cell transplant might be complicate and not completely safe, because it potentially involves hearing damage [77].

Likewise, glial cells reprogramming (i.e., including glial-like supporting cells, SCs or satellite cells) to HCs or SGNs, respectively, is an alternative approach that deserves consistent investigation in the coming future. A paper by Hurley and collaborators [82] suggested that SCs in the auditory nerve might revert to nonmyelinating phenotype in response to HL, exhibiting greater survival trait than SGNs. More recently, other observations revealed that the auditory SCs proliferate after injury, reprogramming and differentiating into SGNs. Indeed, the SCs double positive for the transcription factors Sox2/Sox10 started to proliferate and became neurons [83]. This differentiation was boosted with growth factors (i.e., EGF and bFGF), in combination with valproic acid, indicating a new promising pharmacologic approach for functional recovery and SNHL therapy [83].

To date, therapies for peripheral neuropathies are insufficient, so much that there is no effective treatment for the most common form of CMT1A, and its management remains supportive and symptomatic. Numerous drugs have been proposed to improve the PMP22 overexpression seen in CMT1A [84], with the most clinically advanced being PXT3003 [85]. However, several efforts have been also focusing on emerging gene-silencing approaches, all of which, unfortunately, are still at the preclinical level [84, 86]. Expectedly, future therapies for CMT and, in general, for peripheral neuropathies could be an indirect but alternative approach for treating all those cases of mild HL and HHL.

## Vestibular schwannoma

The VS, also named acoustic neurinoma (see Fig. 2B), is a benign SC-derived tumor of the vestibulocochlear nerve (VIII cranial nerve), coming along the internal auditory canal or the cerebellopontine angle [87]. Histologically, the VS is mostly composed by SCs, showing some typical microscopic features: biphasic architecture (Antoni A and Antoni B patterns), nuclear palisading (Verocay bodies), fibrous capsule with displaced parent nerve and degenerative signs, such as hyalinized vessels, nuclear pleomorphism, hemosiderin deposition [88]. VS can be classified into two forms: sporadic unilateral and those associated with NF2-related schwannomatosis (NF2), which are generally bilateral. VS account for approximately an incidence of 10.4 per million per year [89] and constitute 8% of all benign intracranial tumors, whereas the sporadic form represents up to 95% of all VSs [87]. In recent years, the incidence of VS has increased, given the technological advancement of diagnostic imaging techniques [90]. As a result of earlier detection, the tumor size at diagnosis decreased from 3 cm to the median size of 1.6 cm [90], allowing a reliable and safer prognosis.

**Fig. 3:**
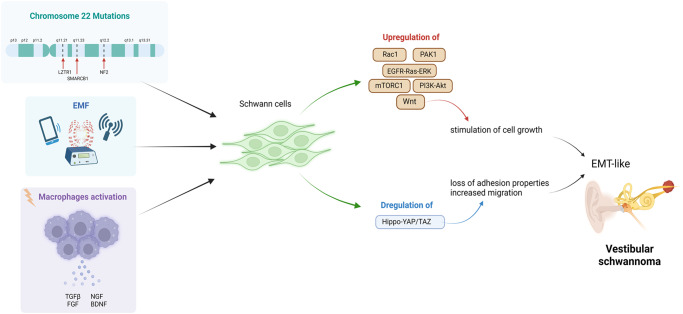
Vestibular schwannoma (VS) pathogenic mechanisms. Genetic mutations in the neurofibromin 2 gene (coding for the protein merlin), but also in other genes (e.g., LZTR1, SMARCB1, or COQ6, RAD54B) cause VS. Also the exposure to electromagnetic field (EMF) and macrophages activation, potentially, might be cause of VS. From a biomolecular point of view, these triggers induce upregulation and activation of some signaling pathways in Schwann cells (SCs), such as Rac1, PAK1, EGFR-Ras-ERK, PI3K-Akt, mTORC1, and Wnt, concomitant with a de-regulation of the Hippo-YAP/TAZ signaling. As a result, SCs growth and migrate, giving the oncotransformation into VS.

**Fig. 4:**
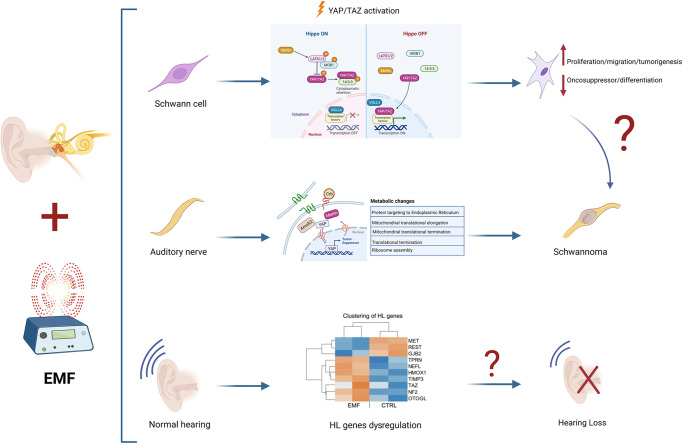
Exposure to electromagnetic field (EMF) might potentially induce changes in the Schwann cells (SCs) of the auditory nerve. Findings obtained from in vitro experiments in rat, even limited by the lack of corresponding data in humans, suggested that SCs exposed to the EMF showed modification in cell proliferation, migration, and differentiation, mediated through an activation of the Hippo transcriptional co-activators YAP/TAZ [114, 115]. Generally, the protein complex YAP/TAZ shuttles between the cytoplasm and the nucleus (see upper part of the figure). When Hippo is on, the oncosuppressor merlin activates the kinase LATS1/2, that in turn phosphorylate YPA/TAZ causing its cytoplasmic retention; thus, the gene expression is inhibited. When Hippo is off, YAP/TAZ moves into the nucleus, links some transcription factors and stimulates the gene expression. When SCs are exposed to EMFs, other proteins downstream merlin, mostly involved in cell polarity, such as Amotl2 and Crb (see the text) are modulated, determining the loss of SCs adhesion and the increase in migration [115]. In parallel, changes in metabolic pathways related to translation and mitochondrial activities, were observed [116]. Notably, the EMF exposure of SCs induces the gene expression alteration of some other genes involved in hearing loss, such as NEFL, TPRN, HMOX1, OTOGL, GJB2, and REST (see the text).

Several mechanisms and factors could at the base of VS onset and progression (see Fig. 3). From a biomolecular point of view, the etiology of sporadic VS as well as that of NF2 is ascribable to the loss of function of the protein named merlin. Indeed, the cause for the transformation of SCs into schwannomas is mainly credited to the inactivation of the neurofibromin 2 gene and the resulting loss of its protein merlin [91], which then leads to a clonal expansion of SCs. However, an additional immune-mediated mechanism has been proposed as the major contributor to the differences in growth rate observed in various tumors. This immune-mediated mechanism, in fact, is responsible for the pronounced infiltration of phagocytic macrophages that participate to the proliferative activity seen in VSs [92]. The neurofibromin 2 gene was identified in 1993, naming the coded protein “merlin” as acronym of the first letters of moesin-, ezrin-, radixin-like protein [93]. Merlin is similar to the cytoskeleton-associated proteins known as ezrin-radixin-moesin (ERM), which present two associated domains (ERMAD), the N-terminal (N-ERMAD) and the C-terminal (C-ERMAD) respectively; these domains allow the ERM proteins to interact with each other, thus establishing the folded protein [94]. Merlin is one of the most versatile tumor suppressor proteins, able to integrate different mechanisms that regulate cell adhesion, proliferation, motility and survival [91]. When merlin is present in the unfolded state, its C-ERMAD domain bind to the actin filaments, mediating cell-cell adhesion and promoting tumor growth. The folded state, instead, represents the tumor suppressor form [94], which is translocated to the nucleus where it binds to the E3 ubiquitin ligase CRL4(DCAF1) suppressing its activity, and in turn halting the gene expression and cell proliferation [95]. Merlin normally inhibits the receptor complex ErbB2/3 [96], target for the NRG-1-dependent SCs proliferation and determinant for the development of the SC precursors into immature SCs. Thereby, loss of merlin results in a stimulation of cell growth. Moreover, loss of merlin activates other signaling pathways important for SCs, such as: Rac1, a protein regulator of cell motility, growth and tumorigenesis; PAK1, member of the serine/threonine kinases, involved in NF2 expansion; EGFR-Ras-ERK, PI3K-Akt and the mammalian target of the rapamycin complex 1 (mTORC1), classic pathways involved in SCs development, proliferation and differentiation; the Wnt pathways, which participate in the process of SCs radial sorting [96–99]. Importantly, the Hippo pathway is the major effector downstream merlin in regulating growth (see Fig. 4 for details), and plays a crucial role in cell proliferation, apoptosis, differentiation and development [100]. When Hippo is activated, the LATS1/2 proteins phosphorylate the co-activators YAP/TAZ that remain in the cytoplasm, blocking the gene expression and realizing the tumor suppression. In SCs (see below), the loss of merlin, via Hippo pathway activation, induces SCs changes in proliferation, migration and likely tumorigenesis [101, 102].

In humans, it was highlighted that also the inactivation of other tumor suppressor genes, such as LZTR1, COQ6 and SMARCB1, is correlated to the schwannoma formation [103, 104]. In particular, it was evidenced that the SWI/SNF chromatin-remodeling complex (i.e., the eukaryotic analog of SMARCB1) possesses and important role for the schwannoma development [103, 104]. Similarly, the RAD54 homolog B (RAD54B) gene, normally associated to the repair of damaged DNA, was found to be downregulated in subjects presenting VS and concomitant poor hearing [105].

Not less important, the analysis of the proteomic composition of the perilymph (the extracellular fluid within the inner ear) unraveled some putative changes of tumor-associated HL, thus indicating that unknown toxic substances may be responsible of HL in VS [106]. In accordance, Lysaght and collaborators [107] found some proteins in the perilymph, like μ-crystallin (CRYM), low-density lipoprotein receptor-related protein 2 (LRP2), dermicidin (DCD), keratin 10 (KRT10), cathepsin D (CTSD), super-oxide dismutase 3 extracellular (SOD3), serpin family B member 12 (SERPINB12), 3 (SER-PINB3) and serpin family A member 5 (SERPINA5), that might be proposed as biomarkers of those forms of human VSs that are correlated to the hearing worsening.

### Vestibular schwannoma and hearing loss

Typically, about 10–30% of patients with VS lesions present unilateral high-frequency SNHL [108, 109], although they can also exhibit tinnitus, headache, vertigo, and motor/balance problems [110]. Therefore, it could be hypothesized that the tumoral expansion of SCs, found in VS, may be one of the causes of SNHL. The failure of SCs capacity to dedifferentiate into the stage of repair/myelinating cells is likely due to a heterozygous NF2 mutation either in the neuronal or in the SCs compartment, which in turn leads to the development of VS [111]. Hence, the VS might be the consequence of the loss of crosstalk between merlin-deficient SGNs and SCs [111].

Noteworthy, the VS compresses mechanically the auditory nerve or the labyrinthine artery, producing a mechanical damage and leading to the unilateral SNHL [112]. In accordance to the “failure-of-nerve regeneration” theory [113], the VS preferentially develops in a location prone to the nerve injury, given by compression or physical trauma.

In any case, the onset of HL is frequently unpredictable, since not all VS cause profound HL. In some cases, indeed, very large VS only cause subtle HL, while in other cases very small VS result in very severe HL. Genes expressed by the VS influence the severity of HL. Thus, an accurate genomic analysis of VS can help to define individual tumor characteristics, promoting a better hearing prognosis [87, 114]. For instance, at least three VS genes (i.e., peroxisome biogenesis factor 5-like, PEX5L; RAD54B; prostate-specific membrane antigen-like, PSMAL) seemed strongly associated with HL [105]. In particular, a reduced expression of PEX5L causes peroxisomal dysfunction with pathological accumulation of fat tissue, demyelination and degeneration of the acoustic nerve [105].

The surgical treatment, even representing the most common approach among the therapies for VS (see chapter below), should be considered as a high-risk morbidity for HL. In parallel, other “physic” and “mechanical” challenges to the auditory system, such as loud and/or chronic noise exposure, represent other risk factors for the development of VS and SNHL. Indeed, these harmful stimuli procure mechanical and electrophysiological signs of nerve damage [24]. Meanwhile, signs of cochlear injury may be due also to vascular occlusion and/or compression, biochemical changes or demyelination [115].

HL has been observed even in non-growing tumors anyhow, so great that the correlation between tumor size and HL suggested that mechanical compression could not be the unique cause of SNHL. Indeed, VS may cause HL also for the concomitant secretion of paracrine toxic substances into the inner ear or into the cochlear nerve [116, 117]. Hence, the tumor microenvironment sustained by infiltrated immune cells (i.e., for the most part M2 macrophages) leads to intratumoral inflammation, playing a key role in VS growth [118]. TGFβ, FGF, NGF, BDNF are an example of some of growth factors released in the tumoral milieu [79–81]. Tumor necrosis factor alpha (TNFα) was indicated as an ototoxic factor, secreted by VSs, which levels were significantly correlated with HL [119]. Also the vascular endothelial growth factor (VEGF) expression is significantly higher in VS than in normal auditory nerve [120, 121], and was associated with HL. This suggested that, at least partially, VS growth is achieved by promoting intratumoral angiogenesis. Finally, as previously reported, an high amount of proteins in the perilymph, such as CRYM, fibronectin 1, CTSD, or LRP2, represents a set of HL biomarkers in VS [107].

In addition, a number of other environmental factors, including the exposure to electric or electromagnetic fields (EMFs) emitted by electronic devices (e.g., domestic appliances, notebooks or mobile phones), has been also suspected as potential risk factor for human health [122, 123], likely including VS onset and growth (see Fig. 4). In the last decades, scientific studies, empirical observations, and patient reports clearly indicate interactions between EMFs exposure and health problems although in the same time the general susceptibility to environmental factors, like EMFs, has been frequently neglected [122]. This claim correlated to the strong development of new technologies, such as the rapid increase in mobile phone use, which, indeed, has raised some safety concerns in terms of tumor incidence for the worldwide population. Epidemiological studies performed in human cohorts, like case-control and case-case analysis, showed a consistent association between tumors and mobile phone use, with a greater correlation to tumor growth rather than to incidence [123–126]. There was also a raising evidence that long-term exposure to certain EMFs could be a risk factor for some diseases, like the Alzheimer’s disease, amyotrophic lateral sclerosis and male infertility [122, 127]. However, this sensitive issue needs future extensive investigations to reach consensus on the cause-effect correlation. Presently, most of the data are epidemiologic and poorly supported by in vivo/in vitro experiments. The few in vivo outcomes from the scientific literature, indeed, are controversial, given the difficulty to compare experimental protocols (e.g., humans vs. rodents or EMF intensity vs. frequency, exposure time, etc.). The international scientific community has been proposing the more prudent hypothesis of “electromagnetic sensitivity” state, to indicate occasional symptoms in humans, including headaches, concentration difficulties, sleep problems, depression and fatigue due to EMFs exposure [128].

### Schwann cell, environmental challenges, and vestibular schwannoma

It was already highlighted that the involvement of SCs in tumor is multifaceted. Some novel evidences indicated that the SCs are likely to play important roles in initiating the tumoral innervation, then contributing to the microenvironment that promote and maintain the tumor development. Of note, in pancreatic and colon cancer, SCs can migrate toward the cancer cells, colonizing neoplastic sites before the onset of cancer invasion [129]. A direct contact between differentiated SCs and cancer cells is then necessary to stimulate their invasion; the adhesion protein NCAM1 (Neural Cell Adhesion Molecule) mediates this interaction [130].

As described above, it is expected that clonal-expanded SCs forming the VS might be responsible of SNHL. The Hippo pathway is the major effector downstream merlin in regulating SCs growth and likely VS (see Fig. 4 upper part for details). Notably, the Hippo transcriptional co-activators YAP/TAZ are essential regulators of peripheral nerve development and myelin maintenance [131, 132]. The developing SCs, therefore, require YAP/TAZ to enter S-phase and to proliferate; in so far, in their absence the nerve fails to generate sufficient SCs for a timely axonal sorting. Furthermore, SCs require YAP/TAZ to differentiate and to upregulate the early-transcription factor Krox20, so that without them the SCs completely fail to myelinate [133].

In SCs the Hippo-mediated mechanisms, involving YAP/TAZ, may be modulated by mechanical stimuli, including the EMF microenvironment (see Fig. 4 for details). In this regard, even considering the limitations of in vitro studies, it was recently suggested that Hippo-YAP/TAZ are involved in the SC alterations occurring after the chronic EMF exposure. In particular, a low-frequency and low-intensity EMF was used (50 Hz and 0.1 Tesla). Although these observations were not supported by other data from in vivo rodent models, likely suggest a potential participation of the EMFs to the nerve tumorigenesis and schwannoma development [101, 102]. In detail, merlin, YAP, as well as other proteins involved in cell polarity, such as Amotl2 (angiomotin like-2 protein) and Crb (Crumbs homolog) protein complex (including proteins 1, 2, and 3), showed a decreased gene expression, suggesting an impairment in tight-junction complex stability, leading to the loss of adhesion properties and to the increase in SCs migrating capability [102].

Human SC lines exposed to a model of chronic EMF revealed modification in cell proliferation, in parallel with intracellular signaling and metabolic pathway changes (Fig. 4), mostly related to translation and mitochondrial activities [134]. Importantly, the expression of some genes known to be involved in the most important forms of SNHL [135, 136], such as NEFL (a neuron-specific intermediate filament essential for the radial growth of axons), TPRN (taper-in), HMOX1 (heme-oxygenase1), OTOGL (otogelin-like protein), GJB2 (Cx32) and REST (a DNA-binding protein that complexes the histone deacetylases), appeared unregulated following the chronic EMF exposure (Fig. 4). Therefore, it was argued that, at a preclinical stage, the EMF exposure might promote the transformation of VS cells, contributing to HL onset or progression. Overall, even considering the limitations, it can be hypothesized that the chronic EMF exposure represents a kind of second hit, affecting the SC development in vulnerable human subjects, specifically those bearing NF2 mutations or changes in merlin expression, and predisposed to develop VS and subsequent HL.

### Therapies for vestibular schwannoma

Despite VSs are clinically regarded as benign tumors, and apparently at low-grade of invasiveness, some severe complications, including irreversible SNHL and facial nerve paralysis, must be considered. Therefore, the pathology itself and the treatment thereof are associated with a significant morbidity. On this basis, the VSs treatment represents a compelling medical need, still challenging and partially unresolved. However, the surgeon must balance and weigh the risk of symptoms worsening versus the potential surgical complications.

The majority of VS usually remain clinically stable and do not require interventional procedures [137]. The approach for most patients with very small VSs (less than 1 cm), indeed, is the “watch and rescan” strategy, simply based on imaging follow-up [137]. In any case, various therapeutic approaches exist to treat VS patients (see Table 2), including the gold standard surgery or the radiotherapy [138]. With the advances of endoscopy for ear surgery, some technical approaches for the management of VSs have been adopted, so that microsurgical resection via retrosigmoid, translabyrinthine or middle cranial fossa are frequently performed. However, due to the common surgical risk of HL and facial weakness, there is growing interest on the use of radiotherapy. This approach may be superior to microsurgery for the management of small and medium-size (less than 3 cm) VSs, giving better hearing and facial outcomes [139, 140], although it is not without riskiness. Therefore, HL, facial nerve dysfunction, post-operative headache, and cerebrospinal fluid leakage may be the consequences of surgery [137].

**Table 2:** Therapeutic approaches and pharmacological therapies for VS.

Vestibular schwannoma		
Therapy	Target	Possible mechanism
Watch and scan	Small tumors (<1 cm)	
Surgery	Larger tumors with acoustic nerve compression	Tumor removal
Radiation	Small lesions	Reduces tumor volume and preserve the nerve’s function
Lapatinib	ErbB family protein inhibitors	Inhibits the tyrosine kinase activity associated with two oncogenes, EGFR and HER2/neu
Ponatinib	PDGFR family proteins	Inhibition of cell proliferation, migration and invasion
Bevacizumab	VEGFR	Inhibition of VEGF-R-mediated angiogenesis
Crizotinib	HGFR	Inhibitor of receptor tyrosine kinases including ALK, HGFR and RON.
OSU-03012/AR12	PI3K/AKT pathway	Inhibitors of AKT signalling
OSU-HDAC42/AR42		
Erlotinib	EGFR-Ras-ERK	Inhibits tumor proliferation
Rapamycin	mTORC1	mTORC1 inhibitors
Everolimus		
AZD2014 + Dasatinib	mTORC 1/2	Inhibit cell growth and proliferation
CXCR4 inhibitor	CXCR4	CXCR4 inhibitor
Mifepristone	*NF-κB*	Decreases cell metabolism and proliferation

However, some pharmacological therapies have long been suggested (see the most challenging in Table 2), even not in clinical practice yet. Emerging treatments for VS are mostly addressed to target SCs, restraining the molecular pathways downstream merlin and/or reducing tumor growth. In this light, bevacizumab, everolimus and erlotinib are the most promising agents. Bevacizumab is a monoclonal IgG1 antibody against VEGF, a modulator of angiogenesis and tumor growth [116, 141]. Everolimus is an mTOR inhibitor, which is believed to inactivate the mTOR pathway dependent by the merlin loss of function [142, 143], while erlotinib is an oral EGFR-Ras-ERK inhibitor, targeting the tumor proliferation [144]. Very recently, Sagers and collaborators [145] demonstrated that the combination therapy with mTORC1/2 inhibitor AZD2014 and the tyrosine kinase inhibitor dasatinib may constitute a novel therapeutic strategy for VS. Importantly, because SCs seem to possess a diriment role in VS pathogenesis, they might represent a promising therapeutic target for addressing novel cell and gene-based therapies.

## Conclusion

The role of SCs in acoustic impairment and in HL pathogenesis was insufficiently investigated so far. Although some observations suggested their involvement in VS pathogenesis, and consequently in HL, the molecular mechanisms controlling such transformations are still partially unraveled and should be further investigated. Here we highlighted the basic knowledge and proposed novel triggers (likely the environmental factors) for the SCs in HL and VS, adding more complexity to the potential correlation among SCs oncotransformation, VS development, HL onset and progression.

Almost all studies associating the SCs transformation and the VS onset to the EMF exposure are epidemiologic, whereas very few pre-clinic in vitro/in vivo studies concerned with this issue. For instance, one study suggested a weak increase in the proliferation rate but not substantial morphological alterations in SCs exposed to EMF [146], whereas differentiation or myelination was not investigated. In rat, continuous exposure to 900 MHz EMF for 1 h, through adolescence, caused oxidative injury, apoptosis, and thickening in the sciatic nerve, even if SCs appeared morphologically normal [147]. Paradoxically, a kind of ultra-low-frequency EMF (15–20 Hz) have been proposed as reliable therapy promoting nerve regeneration [148–150]. Therefore, it can be argued that EMF frequency (ultra-low 50–60 Hz is the typical frequency of electric lines and domestic appliances, while ultra-high 900 MHz is for radio and tv channels) is one of the cruxes for potential in vivo effects. Unrolling the ball of the EMF hazard, the necessary steps for further investigation should rely on a set of objectives: (1) to compare different species, tissues and target organs (e.g., central versus peripheral nerves); (2) to discriminate among different protocols (pulsed or continuous), frequencies and intensities of the applied EMFs, that is different electric/electronic devices (e.g., appliances, notebook, mobile phone, screens); (3) to analyze acute versus chronic exposure times; (4) to identify the cellular and molecular mechanisms downstream the EMF challenges.

In conclusion, even the issue is still controversial, the comprehension of the pathways regulating the biomolecular behavior of SCs could encourage new investigations, and potentially may be the harbinger for the development of new therapies. In this light, we emphasize the hypothesis of cell-based approaches or bioengineering strategies to reprogram the peripheral neural stem cells, potentially helpful as innovative therapies for the hearing impairment. Overall, combining the biomolecular bench-side approaches to the clinical bedside practice may be helpful not only to the treatments but, importantly, also for the prevention, diagnosis and prediction of VS and HL comorbidity.

## Data Availability

All data are available.
